# Cloning of the surface layer gene *sllB* from *Bacillus sphaericus* ATCC 14577 and its heterologous expression and purification

**DOI:** 10.3892/ijmm.2012.890

**Published:** 2012-01-19

**Authors:** YU-BAO CUI, YING ZHOU, WEI-NA LIU, QING-WEN CHEN, GUI-FANG MA, WEI-HONG SHI, YUN-GANG WANG, LI YANG

**Affiliations:** 1 Department of Laboratory Medicine, Yancheng Health Vocational and Technical College, Yancheng 224006, Jiangsu Province; 2 Huainan Bank of Blood, Huainan 230007, Anhui Province, P.R. China

**Keywords:** surface layer, *sslB*, cloning, gene expression, bio*-*informatics

## Abstract

A cDNA fragment encoding the S-layer protein SllB cloned from *Bacillus sphaericus* ATCC 14577 was expressed on the surface of *E. coli* BL21 (DE3) cells and confirmed by the square lattice structure at the nanoscale level. The amplified gene fragment designed with PCR primers from a specified reference sequence (GenBank accession no. AJ849550) showed a high degree of sequence identity with the known sequences for S-layer protein. The best alignment scores were seen in *B. sphaericus* strains JG-A12 and NCTC9602, which code for a pre-form protein with a predicted cleavage site located between the two alanine residues 31 and 32. After this signal peptide sequence was removed, the mature protein had a molecular mass of 116.2613 kDa and a theoretical pI of 5.40. Further bioinformatic analysis revealed three S-layer homology (SLH) domains in the N-terminus of the mature protein, positioned at the 1–61, 63–128 and 137–197 residues. The mature S-layer protein was composed of alpha helices (24.86%), extended strands (27.01%), and rich random coils (48.13%). Bioinformatics-driven characterization of SllB may provide scientific evidence for further application of this gene in the fields of nanobiotechnology and biomimetics in the future.

## Introduction

Crystalline surface layers, also known as S-layers, cover the outermost envelope layers exposed to the extracellular environment. These layers are composed of a single protein (glycoprotein) species with the ability to assemble into two-dimensional arrays in solution, on solid supports, and on various planar lipid membranes during all stages of cell growth and cell division (1,2). S-layers serve a broad spectrum of functions (e.g., protective coats, molecular sieves, molecular and ion traps, cell adhesion mediators, attachment structures, and virulence factors in several pathogenic organisms (3), and they have recently been identified in hundreds of different species of bacteria and archaea (4–6).

The capability of S-layers to reassemble into crystalline arrays provides the basis for their use in the field of nanobiotechnology and biomimetics in basic and applied research, including both life and non-life sciences. Many studies have demonstrated that foreign peptide sequences can be fused without disturbing their self-assembly capabilities (7–9). Since recombinant S-layer proteins and fusion proteins can be successfully produced in heterologous expression systems, genetic manipulation opens new avenues for the use of S-layer protein self-assembly systems. Moreover, attention has also been drawn to the cloning and characterization of genes encoding S-layer proteins. Currently, more than 50 sequences of S-layer genes are available in public databases (10).

*Bacillus sphaericus* is a gram-positive, rod-shaped, spore-forming bacterium. Some strains of *B. sphaericus* are harmless toward insects (11), whereas other strains produce a type of protein that acts as a larvicidal toxin with detrimental effects against the larva of the Wyeomyia mosquitoes. Since this has drastically reduced this mosquito population, *B. sphaericus* is now used worldwide in integrated mosquito control programs (12–14). Previous reports described S-layer proteins in some nontoxic strains of *B. sphaericus* NCTC9602, JG-A12, C3-41, P1 and CCM2177 in detail (8,15). In both JG-A12 and NCTC9602, the chromosomal S-layer protein genes are followed by a newly identified putative insertion element comprised of three open reading frames (ORFs), which encode a putative transposase. This integrase or recombinase is a protein containing a DNA binding helix-turn-helix motif, as well as the S-layer-protein-like gene copies, sllA (NCTC9602) or sllB (JG-A12) (15). To construct chimeric S-layer fusion proteins incorporating biologically active sequences without hindering the self-assembly of the S-layer proteins on surface and in suspension, we tried to amplify the gene fragments encoding the S-layer from *B. sphaericus* ATCC 14577. We failed to do so with the primers designed according to the published sequence *sbpA* (GenBank accession no. AF211170), which encoded the S-layer protein identified in *B. sphaericus* CCM 2177. Unexpectedly, a gene fragment identical to the *sllB* gene, encoding S-layer protein SllB from *Bacillus sphaericus* JG-A12, was amplified. In current studies, S-layer protein genes have been cloned from *Bacillus sphaericus* ATCC 14577 with the primers designed based on the *sllB* gene (GenBank accession no. AJ849550) in *Bacillus sphaericus* JG-A12 and its expression in *E. coli* BL21 (DE3).

## Materials and methods

### Stains, plasmids, and culture conditions. Bacillus sphaericus

ATCC 14577 was provided by the Agricultural Culture Collection of China (ACCC). It is routinely grown in nutrient broth (NB) medium consisting of 5 g peptone 1^−1^ and 3 g meat extract 1^−1^. pMD19-T Simple Vector (code no. D104, Takara Biotechnology Limited Company, Dalian, China) and pET28a (+) (kit lot no. N72770 Novagen, Germany) were used for the cloning and expression, respectively. *Escherichia coli* JM109 competent cells (code no. D9052, Takara) used in cloning; and *E. coli* BL21 used in expression (DE3, Stratagene, USA), were cultivated at 37°C on agar plates and in broth medium.

### Preparation of sllB cDNA and PCR

Genomic DNA of *B. sphaericus* ATCC 14577 was prepared, with the MiniBEST Bacterial Genomic DNA Extraction kit Ver.2.0 (code no. DV810, Takara) according to the manufacturer's instructions. According to the published sequence (GenBank accession no. AJ849550), the oligonucleotide primers were designed with specific sequences, 5′-GGATCCATGGCTAACCAACCAA AGAAATAC-3′ (forward) and 5′-CTCGAGTTATGGAG TAGGCTTTACTGTAATAG-3′ (reverse). These contained a *Bam*HI site and a *Xho*I site at their 5′ ends (underlined), respectively. Using genomic DNA from *B. sphaericus* ATCC 14577 as the template, the *sllB* gene encoding S-layer was amplified by PCR. The reaction system was prepared with the PrimeSTAR^®^HS DNA Polymerase with GC Buffer (code no. DR044A, Takara), and the total reaction mixture contained 1 μl of genomic DNA, 25 μl of 2X PrimeSTAR GC (Mg_2_+plus) Buffer, 4 μl of dNTP mixture (25 mM each), 1 μl each of the forward and reverse primer (20 μM each), 0.5 μl of PrimeSTAR HS DNA Polymerase (2.5 U/μl) and 17.5 μl of dH_2_O. PCR conditions included an initial incubation at 94°C for 3 min, followed by 30 cycles at 98°C for 10 sec, at 55°C for 15 sec and at 72°C for 3 min. After a final incubation at 72°C for 10 min, 5 μl of the amplicons were analyzed by agarose gel electrophoresis (1.0%) and visualized with ImageMaster^®^ VDS.

### sllB cDNA cloning, sub-cloning and sequencing

The PCR-amplified DNA was recovered from the gel with Agarose Gel DNA Purification kit ver. 2.0 (code no. DV805, Takara), and then a poly-A tail was added with DNA A-Tailing kit (code no. D404, Takara). The poly-A tailed product was then cloned into the simple vector pMD19-T (code no. D104, Takara). Then, *E. coli* JM109 (code no. D9052, Takara) was transformed with the recombinant plasmid pMD19-T-*sll*B and positive clones selected by blue/white screening on Luria-Bertani (LB) plates. The inserts were confirmed by restriction enzyme analysis using *Bam*HI and *Xho*I and then sequenced using Primer pMD18F, Primer pMD18R, seqF1 (5′-TTCGCTTCATTCTTATA CCG-3′), seqF2 (5′-ACTTACAAACACCCAGAAAC-3′), seqF3 (5′-CAAAGCGGTAAAGATGCAA-3′) in an ABI PRISM™ 377XL DNA Sequencer (Takara). After alignment of the sequenced results and the reference sequence, a recombinant pMD19-T-*sllB* plasmid with the most homologous reference sequence was digested with *Bam*HI and *Xho*I. To release the *sllB* fragment, the Agarose Gel DNA Purification kit ver. 2.0 (code no. DV805, Takara) was used. Utilization of the DNA Ligation kit (code no. D6023, Takara) allowed creation of pET28a(+)-*sllB* via sub-cloning of the *sllB* cDNA fragment into the expression vector pET28a(+). Competent *E. coli* JM109 cells were transformed with pET28a(+)-*sllB* plasmids, positive clones were selected by blue/white screening, and then confirmed by restriction enzyme analysis with *Bam*HI and *Xho*I.

### Expression, western blotting and purification of sslB in E. coli BL21 (DE3)

A pET28a(+)-*sllB* plasmid was prepared using the MiniBEST Plasmid Purification kit ver. 2.0 (Takara, no. DV801A), from which 0.5 μl was used to transform 100 μl of *E. coli* BL21 (DE3, Stratagene). The *E. coli*BL21 cells carrying pET28a (+)-*sllB* were grown overnight at 37°C on LB plates containing 50 μg/ml of kanamycin. A single colony was inoculated into 5 ml LB containing 50 μg/ml of kanamycin and then cultured at 37°C. Isopropyl-β-D-thiogalactopyranoside (IPTG) was then added (50 μl of a 100 mM stock, final concentration 1 mM) for 3 h to induce the *tac* promoter. The *E. coli* cells were pelleted by centrifugation, resuspended in PBS buffer (200 μl/tube), and ultrasonicly disrupted. One aliquot (50 μl) was harvested to represent whole cell lysate. The remaining lysate was centrifuged to separate the supernatant and pellet, which contained soluble and insoluble proteins, respectively.

The whole cell lysate, soluble protein fraction, and insoluble protein fraction (10 μl each) were subjected to sodium dodecyl sulfate-polyacrylamide gel electrophoresis (SDS-PAGE, 12.5% polyacrylamide gel), followed by CBB-R250 staining. After electrophoresis, the proteins were transferred (39 mA, 80 min) to a PVDF membrane (Tiangen Biotech Co. Led, Beijing, China) and incubated with blocking buffer (1.5% of BSA, 20 mM Tris-HCl, pH 8.0, 150 mM NaCl and 0.1% Tween-20). The membrane was then incubated at 4°C overnight with 1:1000 primary Penta-His antibody (Qiagen, Germany) primary antibody, followed by incubation at room temperature for 1 h with 1:1000 horseradish peroxidase HRP-rabbit anti-mouse IgG (H+L) (Zymed Laboratories, USA). Proteins were then visualized with 1 min exposure to 1 ml of TrueBlue peroxidase substrate (Kirkegaard & Perry Laboratories, Gaithersburg, MD). The purification of the fusion protein products expressed by recombinant *E. coli* BL21(DE) cells was performed using His·Bind kits (Novagen), following the provided protocol, and then the purified proteins were analyzed by SDS-PAGE.

### A transmission electron microscopic observation on E. coli BL21 cells containing pET28a(+)-sllB

The *E. coli* BL21 cells containing pET28a(+)-*sllB* were analyzed by electron microscopy (JEM-2010/INCA Oxford, Japan Electronics Co., Ltd./UK Oxford Company) at the instrumental analysis center of Shanghai Jiao Tong University. The 100 μl precipitate of *E. coli* BL21 cells containing pET28a(+)-*sllB* was harvested by centrifugation, then fixed by glutaraldehyde (2.5%) for 1 h. After two washings, the pellet was resuspended with 50 μl of PBS, followed by the addition of 5 μl of 2% phosphotungstic acid. One hour later, the pellet was washed twice more and resuspended in 50 μl of PBS. Finally, 20 μl of the final product was placed on copper mesh, allowed to dry, and observed under the lamps.

### Bioinformatic analysis of S-layer genes and proteins

The sequenced results were edited to remove vector sequences and extra restriction sites, and then put into BLAST (Basic Local Alignment Search Tool) on GenBank to determine the identity of homologous sequence. The ORF was obtained using the ORF finder on the NCBI (National Center for Biotechnology Information) website. The amino acid sequence of the S-layer was obtained using Translate Tools in the ExPaSy web server, its physicochemical properties were analyzed by ProtParam Tools, and the signal peptide sequence examined by SignalP 3.0 software, hydrophilicity was analyzed by ProtScale tools, secondary structure by GOR4.0, and the functional site by InterProScan, ScanProsite, PPSearch and PROSITE software.

## Results

### Cloning and sequencing of sllB cDNA

**Figure 1:**
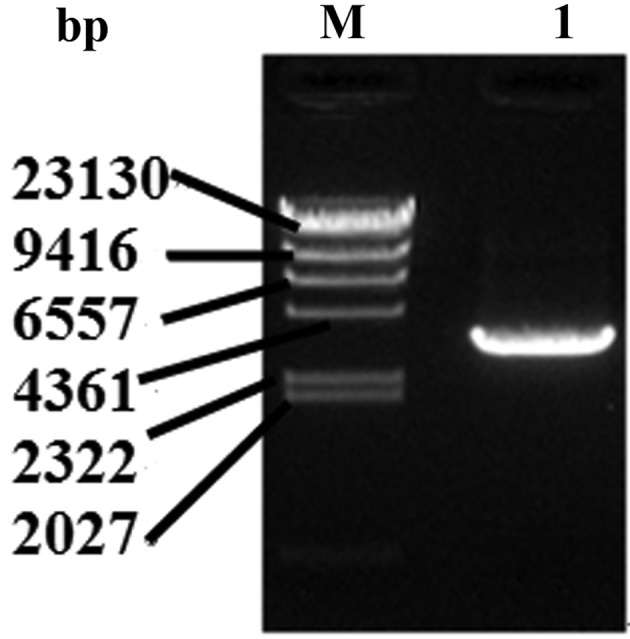
The PCR products of *sllB* from genomic DNA of *Bacillus sphaericus* ATCC 14577 by agarose electrophoresis. Lane M, λ-*Hin*dIII DNA Marker; lane 1, the PCR product.

Using genomic DNA as a template, the gene fragment *sllB* was amplified by PCR with primers designed according to the published sequence (GenBank accession no. AJ849550), then 5 μl of the product was analyzed by agarose gel electrophoresis (Fig. 1). The PCR product was recovered and inserted into a pMD19-T simple vector to create the recombinant plasmid pMD19-T-*sllB*. This was then transformed into *E. coli* JM109 Competent Cells. Positive cells were selected by blue/white screening on LB plates and inserts were sequenced. According to Blastn in GenBank, our sequenced result had a 98.89% identity match with then *B. sphaericus* (strain JG-A12) partial gene for S-layer protein (GenBank no. AJ292965), a 97.57% identity match with *B. sphaericus* (NCTC JG-A12) slfB gene (GenBank no. AJ866975), a 97.46% identity match with *B. sphaericus* (strain JG-A12) sllB gene (GenBank no. AJ849550) for S-layer-like protein, a 91.61% identity match with *B. sphaericus* (strain JG-A12) slfB gene (GenBank no. AJ849549) for S-layer protein, a 90.30 % identity match with *B. sphaericus* (strain NCTC 9602) slfA gene (GenBank no. AJ866974), a 90.30% identity match with *B. sphaericus* (strain NCTC9602) sllA gene (GenBank no. AJ849548) for S-layer-like protein, and a 90.12% match with the *B. sphaericus* (strain NCTC9602) SllA pseudogene (GenBank no. AJ292964). We submitted our sequenced results to GenBank and received the number HM480488. One ORF was identified and the length of the *sllB* nucleic acid sequence was determined to be 3306 bp, after removal of the vector sequence and the added restriction sites.

### Expression and purification of the recombinant protein rS-layer

**Figure 2:**
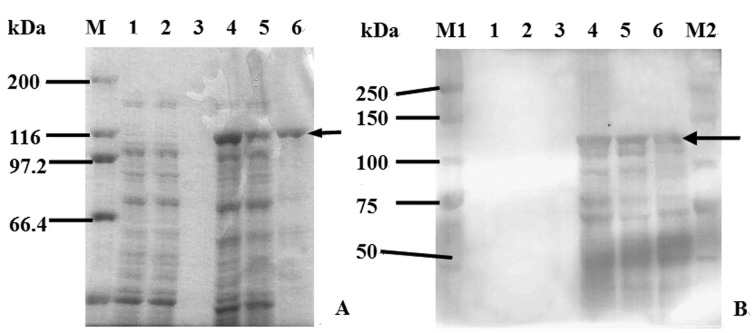
Expression of *sllB* in *E. coli* BL21 cells. (A) SDS-PAGE analysis of rSllB protein; lane 1, the whole cell lysate of *E. coli* BL21 cells containing pET28a(+); lane 2, the supernatant of cells containing pET28a(+); lane 3, the pellet of cells containing pET28a(+); lane 4, the whole cell lysate of *E. coli* BL21 cells containing pET28a(+)-*sllB*; lane 5, the supernatant of cell containing pET28a(+)-*sllB*; lane 6, the pellet of cells containing pET28a(+)-*sllB*; lane M represent Takara Protein Marker (Broad). (B) Western blot analysis of rSllB protein; lane 1, the whole cell lysate of *E. coli* BL21 cells containing pET28a(+); lane 2, the supernatant of cells containing pET28a(+); lane 3, the pellet of cells containing pET28a(+); lane 4, the whole cell lysate of *E. coli* BL21 cells containing pET28a(+)-*sllB*; lane 5, the supernatant of cell containing pET28a(+)-*sllB*; lane 6, the pellet of cells containing pET28a(+)-*sllB*; M, Takara Protein Marker (Broad). Lane M1, Precision plus protein standards; lane M2, perfect protein marker. Note the band pointed with arrows is the recombinant S-layer protein.

**Figure 3:**
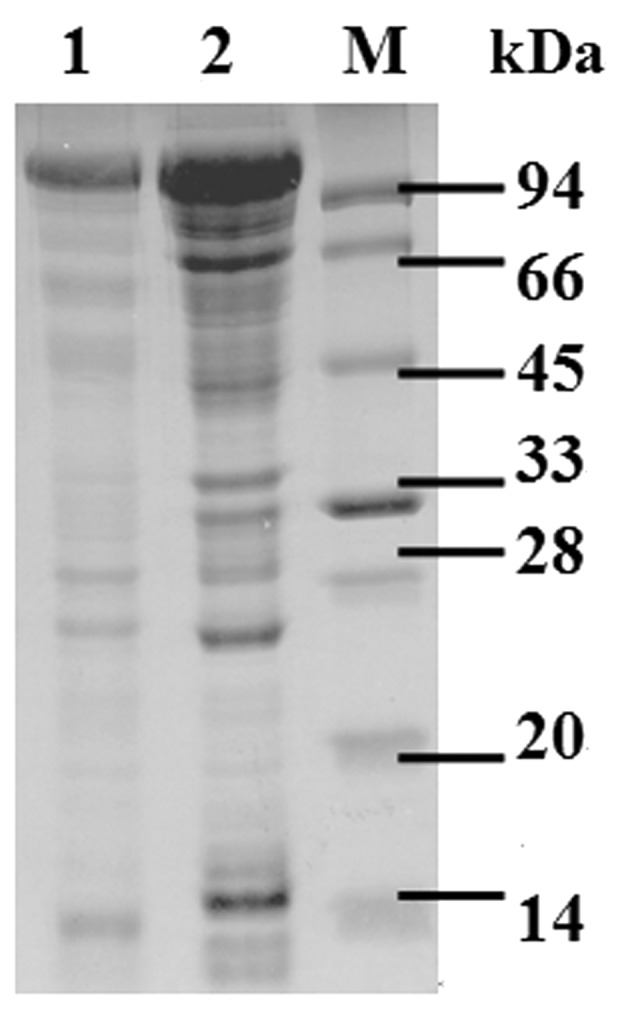
SDS-PAGE analysis of purified protein recombinant SllB in *E. coli* BL21 cells. Lane M: Takara Protein Marker; lane 1, SDS-PAGE analysis of the recombinant S-layer protein before purification; lane 2, SDS-PAGE analysis of the purified recombinant protein.

The *sllB* cDNA fragment was excised from recombinant plasmid pMD19-T-*sllB*, sub-cloned into expression vector of pET28a (+), and confirmed by restriction enzyme analysis. *E. coli* BL21 was transformed with plasmid pET28a (+)-*sllB* and protein expression was induced with IPTG. A single band from SDS-PAGE (Fig. 2A) and western blotting (Fig. 2B) was observed on whole cell lysate, soluble protein fraction, and insoluble protein fraction, confirming the predicted molecular weight for the S-layer protein. *E. coli* BL21 carrying pET28a (+)-*sllB* were recovered from 2L of fermentation solution, collected, ultrasonically disrupted, and centrifuged. The precipitate was washed, dissolved, and filtered, and the supernatant purified by nickel-affinity chromatography, eluted with imidazole solution, and visualized in SDS-PAGE (Fig. 3). The elution fractions were collected, freeze-dried, and 1.8 mg of protein power obtained.

### A transmission electron microscopic observation of recombinant protein on the surface of E. coli BL21 cells

**Figure 4:**
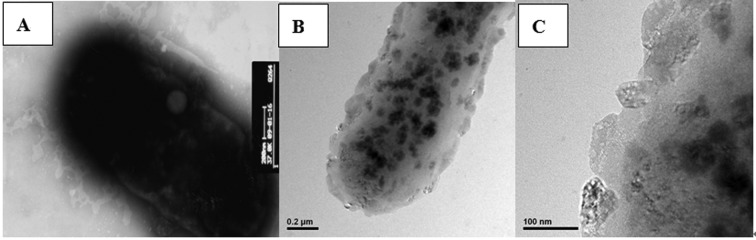
A transmission electron microscopic observation on the *E. coli* BL21 with recombinant protein. (A) Normal *E. coli* BL21 was treated as control. (B) The *E. coli* BL21 cells recombinant S-layer protein. (C) Crystal lattice structures on surface of the *E. coli* BL21 cells recombinant S-layer protein.

A few proteins were observed by electron microscopy on the surface of *E. coli* BL21 cells transformed with pET28a(+)-*sllB* and expressing the recombinant protein rS-layer (Fig. 4). The monolayer consisted of numerous randomly-orientated patches, clearly exhibiting the square lattice structure. The proteins were located on the surface of *E. coli* BL21 cells, and exogenous proteins were also present on *E. coli* BL21 cells.

### Inferred amino acid sequence, physicochemical property, specific motifs and secondary structure prediction

In order to forecast the physicochemical property of recombinant S-layer proteins, the amino acid sequence of the rS-layer was derived from its nucleotide sequence. The deduced pre-protein was found to be composed of 1101 amino acid residues, with a molecular mass of 116.2613 kDa and a theoretical pI of 5.40. By ProtParam, the instability index (II) of the complete prepro-form protein was computed to be 12.25, which classified the protein as stable. The obtained grand average of hydropathicity (GRAVY) was −0.108, demonstrating that this protein was hydrophobic, confirming a previous prediction by ProScale software in the ExPaSy web server. The most likely cleavage site was between amino acid positions 31 and 32.

**Figure 5:**
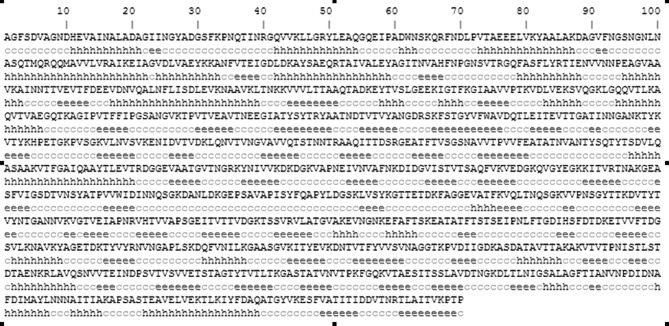
The secondary structure of the mature recombinant S-layer from *B. sphaericus* ATCC14577. H, α-helix; E, extended strand; C, random coil. The results showed the secondary structure of the mature Der f 6 protein was consisted with α-helix (266 aa, 24.86%), extended strand (289 aa, 27.01%), and random coil (515aa, 48.13%).

After this signal peptide sequence was removed, the mature protein was composed of 1070 residues, consisting of Ala (119aa, 11.1%), Arg (20aa, 1.9%), Asn (79aa, 7.4%), Asp (56aa, 5.2%), Gln (35aa, 3.3%), Glu (57aa, 5.3%), Gly (78aa,7.3%), His (5aa, 0.5%), Ile (53aa, 5.0%), Leu (47aa, 4.4%), Lys (77aa, 7.2%), Met (3aa, 0.3%), Phe (37aa, 3.5%), Pro (30aa, 2.8% ), Ser (62aa, 5.8%), Thr (131aa, 12.2%), Trp (3aa, 0.3%), Tyr (36aa, 3.4%) and Val (142aa, 13.3%), with a molecular mass of 113130.6 Da and a theoretical pI of 5.22. Four different software programs, InterProScan, ScanProsite, PPSearch and PROSITE, were used to predict the specific motifs of the recombinant protein. All of these analyses showed consistent results of three S-layer homology (SLH) domains in this mature protein, positioned at 1–61aa, 63–128aa and 137–197aa, respectively. The secondary structure of the mature S-layer was predicted using the GOR4 software and the results showed that 24.86% (266 amino acids) of the protein were alpha helices, 27.01% (289 amino acids) were extended strands, and 48.13% (515 amino acids) were random coils (Fig. 5).

## Discussion

Prokaryotic S-layers have been described as common structures of the cell envelope, with a two-dimensional crystalline array individual subunits formed by an entropy-driven self-assembly event. With the demonstrated feasibility of recombinant S-layer protein production in heterologous expression systems, as well as S-layer fusion proteins incorporating functional sequences self-assembling into monomolecular lattices, new avenues for S-layer protein research have opened up, putting forward the use of S-layer protein self-assembly systems for a broad range of application in the fields of nanobiotechnology and vaccine technology (1–6,10). Identification and characterization of prokaryotic S-layers from different habitats and sources were the main tasks. To our knowledge, NCTC 9602 and JG-A12 are the first strains of *B. sphaericus* found to contain large plasmids encoding silent S-layer protein genes. In the current study, the surface layer protein genes *sllB* were cloned and identified from *Bacillus sphaericus* ATCC 14577, and its complete ORF was 3306 bp. Comparison with known S-layer proteins showed a high degree of sequence homology, especially in the alignment scores of the *B. sphaericus* (strain JG-A12) partial gene for S-layer proteins (GenBank no. AJ292965), *B. sphaericus* (NCTC JG-A12) slfB gene (GenBank No. AJ866975), *B. sphaericus* (strain JG-A12) sllB gene (GenBank No. AJ849550) for S-layer-like protein, *Bacillus sphaericus* (strain JG-A12) slfB gene (GenBank: AJ849549) for S-layer protein, *B. sphaericus* (strain NCTC 9602) slfA gene (GenBank: AJ866974), *B. sphaericus* (strain NCTC9602) sllA gene (GenBank: AJ849548) for S-layer-like protein, and the *B. sphaericus* (strain NCTC9602) sllA pseudogene (GenBank No. AJ292964).

Using SignalP 3.0 software, the predicted cleavage site was found between alanine residues 31 and 32, suggesting that the expressed S-layer proteins *SllB* have identical signal peptides of 31 amino acid residues that are responsible for the protein secretion to the cell surface. After removal of the signal peptide sequence, the theoretical molecular masses of the mature protein were expected to be 113130.6 Da. which was found to be in accordance with the masses determined by SDS-PAGE. A total of 3 SLH domains in this mature protein were predicted to be positioned at 1-61aa, 63–128aa and137–197aa, respectively. They were adjacent to the secretion signal in the N-terminal part, which was reported to be responsible for the non-covalent attachment of S-layer proteins and other proteins to the pyruvylated secondary cell wall polymers (SCWP) of Gram-positive bacteria (16–18). Ryzhkov *et al* reported that the two striking proline residues in the SLH sequence are required for the correct folding of the S-layer protein leaving the utmost N-terminal portion surface-exposed for the attachment to the bacterial SCWP (19). These structural characterizations and distinctions provide useful information for full use of this gene fragment.

In biochemistry and structural biology, secondary protein structure is the general three-dimensional form of local segments of proteins, defined by patterns of hydrogen bonds between backbone amide and carboxyl groups (20). By the GOR IV program, the secondary structure of the mature protein is composed of alpha helices (24.86%), extended strands (27.01%), and random coils (48.13%). In general, the most common secondary structures in proteins are alpha helices and extended beta sheets; however the random coil is not a specific shape but a class of conformations that indicates an absence of a regular secondary structure. The abundance of random coils in *SllB* should be further explored to explain the relationship between the structure and function of this protein.

Heterologous expression of *sllB*, or genetically engineered derivatives, is of high interest for future applications in nanobiotechnology. Therefore, through cloning the *sllB* from *Bacillus sphaericus* ATCC 14577 into the pET28a(+) vector for expression in *E. coli* BL21 cells (DE3), this initial experiment demonstrated the capacity to synthesize this recombinant protein. By electron microscopy, the recombinant proteins were observed on surfaces of *E. coli* BL21 cells, clearly exhibiting the square lattice structure at nanoscale levels. Although only 1.8 mg of protein powder was obtained from a 2L fermentation solution of *E. coli* BL21 carrying pET28a (+)-*sllB* cells, this expression level seems to be similar for *SllB* of *Bacillus sphaericus* JG-A12 (15). The reason for these low expression levels may be associated with biased codon usage (e.g. rare codons for leucine, isoleucine, argine and serine). In the future, recombinant *SllB* should be produced in the *E. coli* expression strains bearing the set of tRNA genes specific for rare codons (19).
